# Psychological factors responsible for low adherence to mask-wearing measures during the COVID-19 pandemic

**DOI:** 10.1192/bjo.2022.603

**Published:** 2022-11-23

**Authors:** Cengiz Kılıç, M. İrem Yıldız, Esra Emekli, Gülhan Gülşen, Anıl Alp

**Affiliations:** Department of Psychiatry, Faculty of Medicine, Hacettepe University, Turkey; and Stress Assessment and Research Centre, Hacettepe University, Turkey; Department of Psychiatry, Faculty of Medicine, Baskent University, Turkey; Department of Psychiatry, Faculty of Medicine, Hacettepe University, Turkey

**Keywords:** Phobias, epidemiology, anxiety disorders, COVID-19, face masks

## Abstract

**Background:**

The COVID-19 pandemic has led to >6 million deaths. Anti-mask movements may decrease the effects of preventive measures. Psychological factors that may be related to anti-mask behaviour are not well researched.

**Aims:**

This study aims to determine the psychological correlates of anti-mask attitudes and behaviour in an online general population sample, focusing on the possible role of claustrophobia.

**Method:**

Data on attitudes and behaviour toward mask-wearing were collected from an online sample of 3709 people. Predictors of both anti-mask attitudes and behaviour were assessed with linear and logistic regression analyses.

**Results:**

Few people (3.3%) were overtly opposed to mask-wearing; mask opposition was more common in men than women. Predictors of negative attitude toward mask-wearing and low adherence to mask-related measures were similar and included male gender, lower education, lower income, being employed, having had COVID-19 and lower COVID-19-related anxiety. Psychopathology measures did not show a prediction, whereas claustrophobia had a significant prediction that was over and above those of other predictors. Avoidance behaviour had similar predictors, except for higher COVID-19-related anxiety.

**Conclusions:**

Although low adherence to mask-wearing during the pandemic was not related to having a mental disorder, it may partly be caused by psychological factors. Those who had a negative attitude also reported lower adherence behaviour, and were characterised by being male, having lower education, being employed and having lower COVID-19-related anxiety; claustrophobia was a strong predictor of attitude. Understanding psychological factors responsible for low adherence may help to decrease morbidity and mortality in future pandemics.

The COVID-19 pandemic is currently one of the most serious global public health concerns. The worldwide death toll from COVID-19 has reached 6.32 million.^1^ Although the introduction of vaccines has significantly decreased the number of new cases and deaths, the pandemic is not yet over and continues to be a major public health concern, especially in countries with limited access to vaccines and underdeveloped healthcare infrastructure. Further, the World Health Organization has warned that other pandemics are highly probable in the near future.^2^ Several studies over the past 2 years have consistently shown that preventive measures, such as mask-wearing, social distancing and hand-washing, have contributed to the fight against COVID-19.^3–6^ It is therefore important to determine factors that decrease adherence to preventive measures, to be able to lower the number of COVID-19 cases and deaths and for future pandemics.

## Factors related to mask-wearing during the pandemic

Among the several preventive measures, mask-wearing deserves special attention because it is the most visible of all preventive measures. It may also be more likely than other measures to be related to psychological factors. Studies on adherence to mask-wearing during the pandemic have shown that men, younger people and less educated people tend to wear masks less often than others.^7–12^ Research by Howard^13^ and Mallinas et al^14^ did not find any differences in adherence to mask-wearing according to gender. Milad and Bogg^15^ reported that there was no link between gender and mask-wearing, although female gender predicted a greater level of guideline adherence. Political leaning is shown to affect adherence to COVID-19 preventive measures.^16,17^ In particular, being politically conservative is associated with low levels of adherence.^12,15,18^ Although no study has addressed anti-mask behaviour directly, studies on adherence suggest that ‘anti-maskers’ are a small minority in most countries.^19^ Psychological or behavioural characteristics that are predictive of adherence to preventive measures are less commonly studied.^20,21^ The few studies that examined such characteristics observed that COVID-19-related anxiety or general health anxiety is associated with a high level of adherence.^7,9,14^ Studies on personality traits^22,23^ suggest that high levels of self-control,^24^ agreeableness, conscientiousness and extraversion, and a low level of neuroticism,^15,25^ are associated with adherence to COVID-19 preventive measures, including mask-wearing.

## Claustrophobia: a probable psychological reason for non-adherence

Among the possible psychological factors that may be linked to low adherence to mask-wearing measures, claustrophobia warrants investigation. Claustrophobia is common in the general population, is usually undertreated and, in severe cases, can cause significant disability.^26,27^ Judging from the large volume of publications from other areas of medicine depicting ‘problem patients’ who would not go into a magnetic resonance imaging chamber or not use oxygen masks properly, it is highly likely that the burden of claustrophobia is greater than observed by mental health professionals.^28,29^ It is intuitive to think that people with claustrophobia would have difficulty wearing face masks, which many would perceive as leading to difficulty breathing – the most significant fear cue (i.e. suffocation) associated with claustrophobia.^30,31^ In fact, a common complaint about face masks is that ‘they make it impossible to breathe’, despite the fact that there is no significant decrease in oxygen saturation in people wearing face masks.^27^ As such, it was expected that there would be many studies on the effects of claustrophobia in lowering adherence to mask-wearing measures during the COVID-19 pandemic. Surprisingly there were none; the only study that examines claustrophobia in the context of the pandemic focuses on the effect of lockdowns on claustrophobia.^32^

The first case of COVID-19 in Turkey was detected on 16 March 2020, and the first COVID-19-related death was reported on 23 March 2020. After the introduction of vaccines in January 2021, the Turkish Government was optimistic that the spread of COVID-19 would be controlled and most COVID-19 restrictions were lifted in July 2021, which led to an abrupt increase in the number of cases and deaths. In Turkey, mask-wearing in public was mandatory between March 2020 and March 2022. Beginning with April 2022, mask-wearing measures were partly lifted, and currently, mandatory mask-wearing is in effect only in hospitals.

In light of the related literature and our own clinical observations, we aimed to study the probable psychological variables associated with low adherence to mask-wearing measures. The topic is important because wearing masks is proven to be protective, even for those that are vaccinated.^33,34^ Further, as most psychological problems are treatable, data collected via such a study could help increase adherence to COVID-19 preventive measures and decrease spread of the virus, hence potentially saving lives. The present study aimed to determine the psychological reasons for opposition to mask-wearing in Turkey, using an online sample. Study variables included COVID-19 experience, COVID-19-related anxiety, claustrophobia severity and self-reported attitudes and behaviour toward mask-wearing mandates. It was hypothesised that low adherence to mask-wearing would relate positively to claustrophobia and negatively to COVID-19-related anxiety.

## Method

### Participants

The participants comprised 3743 volunteers aged ≥15 years and living in Turkey. We recruited participants via advertisements on social media platforms (Facebook, Twitter and Instagram). All those that subsequently contacted us were asked to forward the study link to their contacts, so as to enlarge the study population. The participants’ personal identifying information and email addresses were not collected. Data collection was performed between 1 and 30 April 2021. Thirty-four people who did not wish to report gender information were excluded from analyses. The final sample comprised 3709 people.

### Measures

Data were collected with an online battery of study measures via Google Forms (Google LLC, Mountain View, CA, USA, https://www.google.com/forms/about/). Because of the unavailability of published relevant measures in Turkish, we had to develop measures for self-reported COVID-19-related anxiety, claustrophobia severity and attitudes and behaviour toward government-imposed mask mandates. It is not easy to obtain reliable data on sensitive issues from survey participants, especially if the questions have the potential to make them feel uncomfortable. In fact, mask-wearing in public was mandatory in Turkey during the data collection period, with non-adherence punishable by law. As such, the participants were not directly asked ‘Do you wear a mask in public spaces?’. Instead, indirect or hypothetical questions were used elicit more realistic responses for negative attitudes and behaviour related to mask-wearing.

### Measures of psychopathology

#### COVID-19-related Anxiety Scale

The nine-item COVID-19-related Anxiety Scale (CAS), developed for this study, is used to assess common fears and anxieties regarding possible COVID-19-related negative outcomes in the past month. The instruction was: ‘Please rate the level of distress you felt in the last month about the probable negative outcomes of Covid-19’. Examples for items are anxiety from the possibility of acquiring COVID-19 and anxiety from the possibility of passing COVID-19 to loved ones. Items are answered on a four-point Likert type scale (0, none; 3, extreme). The scale was developed by the researchers for use in pandemic-related research. The total score ranges from 0 to 27. Cronbach's alpha for the scale in the current sample was 0.88; Spearman–Brown split-half reliability score was 0.85.

#### Claustrophobia Severity Scale

The 21-item Claustrophobia Severity Scale (CSS) was developed by the researchers specifically for this study. It assesses lifetime presence of fear of common claustrophobic situations, such as being in an elevator or crowded bus. The instruction was: ‘Some people are extremely anxious or fearful in closed places where they feel escape is difficult, and they avoid such places. Please rate ‘difficult’ if being in such situations is very hard for you, and ‘not difficult’ if not very hard. If you have not ever been in the described situation, rate thinking how you would feel if you had to be in such a situation. We are asking how you feel in general. Therefore, if the difficulty you are experiencing is solely related to the pandemic or to a fear of catching the virus, rate ‘not difficult’. Typical items were getting on an elevator, getting on a crowded bus and sitting in a room with no window. The total score ranges from 0 to 21. Cronbach's alpha for the scale in the current sample was 0.88; Spearman–Brown split-half reliability score was 0.85.

#### History of psychiatric illness

The respondents were asked if they had ever been diagnosed with any of seven common psychiatric disorders (attention-deficit hyperactivity disorder, depression, panic disorder, phobias, post-traumatic stress disorder, obsessive–compulsive disorder and generalised anxiety disorder). The responses were then recoded as 1 (yes) or 0 (no), where ‘yes’ meant any psychiatric diagnosis and ‘no’ meant no diagnosis.

#### Contact with mental health services

Any contact with a mental health professional for psychological problems in the past 12 months was coded as present or absent.

### Measures of attitude toward masks

#### Negative attitude toward mask-wearing

The 35-item Negative Attitude toward Mask-Wearing Scale (NAM) was developed by the researchers for this study to assess attitudes toward mask-wearing in the past month. The instruction was: ‘Below are some statements people who do not want to wear masks in public give as reasons. Please rate each item considering their effect on your willingness to wear masks. Please consider the last month in your ratings’. Typical items were ‘I can't breathe with the mask on’, ‘COVID-19 is not as contagious as we are told to believe’ and ‘I look funny with the mask on’. Each item was coded as 0 (I do not agree), 1 (I agree, but it does not affect my attitude toward masks) or 2 (I agree and that's why I don't want to wear masks). Sum of the responses rated as ‘2’ was computed and referred to as the NAM score. The total NAM score ranges from 0 to 35 and the Cronbach's alpha of the scale in the current sample was 0.94; Spearman–Brown split-half reliability score was 0.90.

#### Mask opposition

A single item on the NAM (mask opposition) was also included to test the performance of the NAM. We asked the respondent a hypothetical question: ‘How would you behave if mask-wearing was not mandatory?’. Response options were 1, ‘I would only wear a mask indoors’; 2, ‘I would never wear a mask’; 3, ‘I would wear a mask only to protect family and friends’ or 4, ‘I would only wear a mask near suspected COVID-19 cases’. We recoded these responses as 1 (‘I would not wear a mask’) or 0 (all other responses).

### Measures of mask-related behaviour

#### Mask Avoidance Scale

The six-item Mask Avoidance Scale (MAS) assesses various behaviours used by the respondents in the past month to specifically avoid wearing a mask. It was developed by the researchers for this study. The items were worded so as to exclude avoidance from other causes. Each item is coded as yes or no and the total score ranges from 0 to 6. The instruction was: ‘Please rate yes if you avoid the situation solely to avoid wearing a mask, and rate no if you are avoiding the situation because of fear of the pandemic’. Typical items were ‘I decreased/stopped eating out to avoid wearing a mask’ and ‘I decreased/stopped using public transport to avoid wearing a mask’. Cronbach's alpha for the scale in the current sample was 0.86; Spearman–Brown split-half reliability score was 0.84.

#### Adherence to mask-wearing measures

We used a single item that asks respondents to make an assessment of their own adherence to mask-wearing measures in the past month, as compared to others. The item was: ‘Please rate the degree to which you comply with the mask-wearing rule compared to others in the LAST MONTH’. The response set was 5 (‘Much more than others’), 4 (‘A little more than others’), 3 (‘Same as others’), 2 (‘A little less than others’) and 1 (‘Much less than others), to give the adherence to mask-wearing measures score (ADM score). Adherence to social distancing, cleaning and lockdown measures were also assessed with similar single-item questions, but were not included in the study's analyses.

### Statistical analyses

Chi-squared and *t*-test analyses were used to compare men and women in the sample in terms of the study variables. Pearson correlation was used to examine bivariate correlations between those variables. Linear regression was used to find out the predictors of mask-related attitudes and behaviour. Finally, logistic regression was used to examine the predictors of mask-opposition. In conducting regressions, variables were entered in three hierarchical blocks to see relative predictive power of each explanatory variable group. SPSS version 26 for Windows was used in all analyses.

### Ethical approval

The authors assert that all procedures contributing to this work comply with the ethical standards of the relevant national and institutional committees on human experimentation and with the Helsinki Declaration of 1975, as revised in 2008. All procedures involving human participants were approved by Hacettepe University Ethics Board for non-interventional clinical research on 23 February 2021 (approval number 2021/04-53). The online form included a consent paragraph that the participant had to read and accept to be able to proceed with filling in the form – a process that was approved by the ethics board.

## Results

### Description of the sample

The study sample differed from the Turkish general population in terms of gender and education.^35^ Mean age of the sample was 36.9 years; there was no gender effect on age. Women are known to respond to online COVID-19 pandemic surveys more often than men. The present study was no exception in this regard, as 66% of the sample was female. In addition, 66% of the sample had a college or higher level of education, compared with 20% in the general population. On the other hand, the COVID-19 polymerase chain reaction test positivity and vaccination rates in the current sample were very similar to those in the general population. At the start of data collection (first week of April 2021), 24.4% of the study participants had received their second COVID-19 vaccine doses (Sinovac). In the first week of April 2021, the Turkish national percentage for receiving two doses was 12%, which is equivalent to 24% among those aged ≥15 years (study sample was limited to those aged ≥15 years). Similarly, the Turkish Government's official rate of total COVID-19 cases among those aged >15 years was very close to the 15.5% noted in the present study. In total, 66% of the participants that reported having been infected with COVID-19 were infected between September and December 2020.

### Clinical variables

Past psychiatric illness as well as mental health service use in the past year were higher among women than men. COVID-19-related anxiety (CAS) and claustrophobia (CSS) scores were also higher in women than men (Table 1). COVID-19-related attitude or behaviour variables did not relate to any other demographic variable (Table 2).

**Table 1:** Study variables by gender

	Male, *n* (%)	Female, *n* (%)	Total, *n* (%)
Employed** (0 = no, 1 = yes)	752 (63.8)	1193 (47.2)	1945 (52.4)
Married** (0 = no, 1 = yes)	741 (62.8)	1216 (48.1)	1957 (52.8)
12-month contact with services** (0 = no, 1 = yes)	110 (9.3)	368 (14.5)	478 (12.9)
Psychiatric history** (0 = no, 1 = yes)	224 (19.0)	686 (27.1)	910 (24.5)
COVID-19 positive (0 = no, 1 = yes)	184 (15.6)	390 (15.5)	574 (15.5)
Loss of family member to COVID-19 (0 = no, 1 = yes)	52 (4.4)	129 (5.1)	181 (4.9)
Mask opposition** (0 = no, 1 = yes)	68 (5.8)	54 (2.1)	122 (3.3)
	Mean (s.d.)	Mean (s.d.)	Mean (s.d.)
Age (years, 15–77)	36.8 (13.9)	36.9 (14.3)	36.9 (14.2)
Education** (1 = primary school, 2 = secondary school, 3 = high school, 4 = university, 5 = postgraduate)	3.94 (.86)	3.84 (.87)	3.87 (0.87)
Negative attitude toward masks** (0–35)	3.49 (7.2)	2.29 (5.6)	2.70 (6.3)
Adherence to mask-wearing rules** (1–5)	4.25 (.99)	4.49 (.80)	4.4 (0.87)
COVID-19-related anxiety score** (0–27)	11.79 (6.7)	13.77 (6.3)	13.1 (6.5)
Claustrophobia severity score** (0–19)	3.44 (3.8)	5.01 (4.3)	4.53 (4.2)
Mask avoidance score (0–6)	1.36 (1.9)	1.49 (1.9)	1.45 (1.9)

**P < 0.01.

**Table 2:** Correlation between study measures

	Age	Education	Income	ADM score	CAS score	CSS score	MAS score	NAM score
Age	1	−0.09**	−0.06**	0.01	−0.05**	−0.01	0.02	0.01
Education	−0.09**	1	0.23**	−0.04*	0.03	−0.05**	−0.07**	−0.07**
Income	−0.06**	0.23**	1	−0.01	−0.05**	−0.06**	−0.02	−0.06**
ADM score	0.01	−0.04*	−0.01	1	0.24**	0.03	−0.04*	−0.41**
CAS score	−0.05**	0.03	−0.05**	0.24**	1	0.23**	0.09**	−0.14**
CSS score	−0.01	−0.05**	−0.06**	0.03	0.23**	1	0.22**	0.17**
MAS score	0.02	−0.07**	−0.02	−0.04*	0.09**	0.22**	1	0.23**
NAM score	0.01	−0.07**	−0.06**	−0.41**	−0.14**	0.17**	0.23**	1

ADM, adherence to mask-wearing measures; CAS, COVID-19-related Anxiety Scale; CSS, Claustrophobia Severity Scale; MAS, Mask Avoidance Scale; NAM, Negative Attitude toward Mask-Wearing Scale.

*P < 0.05, **P < 0.01.

### Correlates of mask-related attitudes

NAM score was higher in men than women (Table 1). NAM score was also higher among the employed. NAM score had a moderate positive correlation with ADM score, weak positive correlations with CSS and MAS scores, and a weak negative correlation with CAS score. COVID-19-related anxiety (CAS score) had weak positive correlations with ADM and CSS scores. Claustrophobia (CSS score) had weak positive correlations with CAS and MAS scores (Table 2). The single-item attitude variable ‘mask opposition’ behaved similarly to NAM: mask opposition was much higher among men than women (5.8 *v*. 2.1%), and among employed than unemployed (3.9 *v*. 2.6%).

### Correlates of mask-related behaviour

Women reported higher adherence than men to mask-wearing measures (as measured by ADM score); no gender differences were found for self-reported avoidance of activities so as to avoid wearing a mask (MAS score) (Table 2).

MAS score had weak positive correlations with claustrophobia (CSS score; 0.22) and negative attitudes (NAM score; 0.23). ADM score, on the other hand, had a moderate negative correlation with NAM score (−0.41), and a weak positive correlation with CAS score (COVID-19-related anxiety; 0.24) (Table 2).

### Predictors of negative attitudes and behaviour related to mask-wearing

Twelve variables were included to examine the independent predictors of negative attitude (NAM score and mask opposition item) and behaviour (mask avoidance (MAS score) and adherence to mask-related measures (ADM score)). Binary logistic regression was used to examine mask opposition, and linear regressions were used for other outcome variables (NAM, MAS and ADM scores). Predictor variables were entered hierarchically in three blocks, to examine the relative strength of predictions. We entered demographics and COVID-19-related variables first, psychopathology variables (psychiatric history, contact with services) second and claustrophobia severity, CSS) last; this design will allow us to see if the prediction of our outcome variables by claustrophobia is over and above the prediction by all other variables.

#### Predictors of negative attitudes toward mask-wearing

Among the first set of predictors of negative attitude toward mask-wearing, predictions by male gender, low education and low income, being employed, having had COVID-19 and lower COVID-19-related anxiety scores were significant (Table 3). Among the second set of predictors (psychopathology variables), past psychiatric illness was a significant predictor. In the final model, claustrophobia was a significant predictor of negative attitude toward mask-wearing, as well as other variables found to be predictors in the previous steps, except past psychiatric illness, which lost its prediction in the full model after claustrophobia was added.

**Table 3:** Predictors of mask-related attitudes and behaviour: linear regression

	NAM		MAS		ADM	
	Standardised *β*	*t*	Standardised *β*	*t*	Standardised *β*	*t*
Age	−0.01	−0.41	0.02	1.29	0.02	0.95
Gender	−0.11**	−6.41	−0.02	−0.98	0.09**	5.50
Education	−0.07**	−3.68	−0.06**	−2.88	0.00	0.04
Income	−0.05**	−3.02	0.01	0.84	0.022	1.35
Marital status	−0.03	−1.49	0.00	0.02	0.01	0.49
Employment	0.06**	2.88	−0.01	−0.69	−0.09**	−4.50
COVID-19 positive	0.03*	2.10	0.01	0.69	−0.04*	−2.48
Loss of family member to COVID-19	0.01	0.34	−0.02	−1.09	0.03	1.70
CAS score	−0.18**	−10.68	0.05**	2.76	0.24**	14.41
Psychiatric history	0.01	0.55	−0.02	−0.88	−0.01	−0.74
12-Month contact with services	0.01	0.61	0.001	0.07	−0.02	−1.30
CSS score	0.22**	13.47	0.22**	12.76	−0.04*	−2.39
*R*^2^	0.08		0.06		0.08	
*F* (d.f.)	27.58 (12,3685)		18.42 (12,3685)		25.99 (12,3685)	
*P-*value	0.000		0.000		0.000	

NAM, Negative Attitude toward Mask-Wearing Scale; MAS, Mask Avoidance Scale; ADM, adherence to mask-wearing measures; CAS, COVID-19-related Anxiety Scale; CSS, Claustrophobia Severity Scale.

*P < 0.05, **P < 0.01.

Predictors of mask opposition found in logistic regression analyses were very similar to those of negative attitude toward mask-wearing: among the first set of variables, prediction of mask opposition by male gender, lower education, being employed and lower COVID-19-related anxiety was significant (Table 4). The two psychopathology variables added at the second step were not predictive. In the final model, claustrophobia predicted mask opposition; variables that were predictors in the previous steps sustained their predictive power.

**Table 4:** Predictors of mask-related attitudes: binary logistic regression

			95% CI for Exp(B)	
	Wald	Exp(B)	Lower	Upper
Age	1.82	1.01	0.99	1.02
Gender	21.48	0.39**	0.26	0.58
Education	5.50	0.75*	0.58	0.95
Income	0.30	1.09	0.81	1.45
Marital status	0.02	1.03	0.67	1.59
Employment	6.06	1.83*	1.13	2.95
COVID-19 positive	0.07	1.07	0.65	1.74
Loss of family member to COVID-19	2.14	1.74	0.83	3.63
CAS score	54.14	0.89**	0.86	0.92
Psychiatric history	0.34	0.86	0.53	1.42
12-Month contact with services	0.02	1.04	0.57	1.90
CSS score	30.45	1.12**	1.08	1.17
*χ*^2^ (12, *N* = 3696) = 127.26, *P* = 0.000				

CAS, COVID-19-related Anxiety Scale; CSS, Claustrophobia Severity Scale.

*P < 0.05, **P < 0.01.

### Predictors of behaviour related to mask-wearing

Among the first set of variables predicting adherence to mask-related measures, female gender, not being employed, not having had COVID-19 and higher COVID-19-related anxiety were significant. The addition of psychopathology variables did not add any new prediction. In the final model, lower claustrophobia score was predictive; predictors in previous steps sustained their prediction (Table 3).

Among the variables added at the first step, mask-avoidance (MAS score) was predicted by lower education and higher COVID-19-related anxiety (Table 3). Addition of psychopathology variables at the second step did not make any changes. In the final model, claustrophobia was a predictor along with predictors at the first step.

## Discussion

Mask-wearing has been demonstrated to be an effective measure for protecting oneself and others from COVID-19 infection.^33^ Therefore, identifying the factors that decrease adherence to mask-wearing is important. Although several social, political and demographic factors have been reported to be associated with low-level motivation or opposition to mask-wearing,^7,9,10^ few studies have examined the possible psychological reasons that are, at least partly, responsible for these associations.^9,14,15^ The present study aimed to discern the reasons. Measuring adherence with COVID-19 preventive measures is a difficult task, particularly because when such measures are mandated by law, it can be difficult to obtain reliable information from study participants. It is also quite difficult to collect accurate information retrospectively, because of the unreliability of human memory. The present study attempted to overcome some of these difficulties by assessing mask-related attitudes and behaviour based on several direct and indirect survey questions.

In the present study, we measured attitude with two different variables: one was a total score of a scale assessing several reasons people present for a certain dislike or low motivation for wearing the mask; and the other was a single item, rating a hypothetical question that we refer to as mask opposition (‘How would you behave if mask-wearing was not mandatory?’). The results show that mask-related attitudes closely parallel adherence behaviour: male gender, being employed, lower education, higher claustrophobia, lower COVID-19-related anxiety and having had COVID-19 related both to negative attitudes and low adherence toward mask-wearing mandates. Mask avoidance, on the other hand, was mainly predicted by claustrophobia scores. Claustrophobia strongly predicted negative attitude toward masks and mask opposition, although it was a weak predictor of low adherence. Taken together, these findings show that although individuals with claustrophobia dislike masks and do not want to wear them and try to avoid wearing whenever possible, they are not excessively disobeying mask-wearing rules (avoidance cannot be equated with non-adherence).

Gender seems to be a central factor in determining attitude and behaviour related to mask-wearing. Our findings show that men are more likely than women to oppose masks. The majority of studies that assessed the effect of gender on adherence to mask-wearing measures reported the same finding.^10,36,37^ Vaccine opposition (those who said they did not and will not get a vaccine shot), which strongly correlated with mask opposition, did not show a gender effect, which is also in line with literature.^4,38,39^

Unlike the present study's sample, in most published studies, adherence to mask-wearing measures is reported to increase with age. Most senior citizens in Turkey were confined (by law) to their homes during the data collection period, which might have limited their need to wear masks. The present study's finding that there is a strong link between a negative attitude toward masks and being employed might be explained by the fact that the participants that were not employed had much more control over mask-wearing, whereas those working outside of the home had to wear masks when commuting and working. Of note, the lockdown measures for employed people were lifted during the data collection period, and working from home is not common in Turkey. In other words, being employed during the study period meant physically going (travelling) to work.

### Limitations

Our study had several limitations. Because of the unavailability of measures available in Turkish, we had to develop most of the study measures ourselves. On the other hand, the scales developed by our team had high reliability figures. Although anonymous data collection may have increased honest reporting, our measures were online self-report questionnaires, which do not elicit mental disorder prevalence. All psychological disorders that could cause discomfort or anxiety related to mask-wearing (and hence low-level adherence) were not assessed. Additional research that includes comprehensive psychological health assessment will more clearly discern all psychopathological factors related to mask opposition. The data collected were cross-sectional and, as such, it was not possible to make causal inferences. The COVID-19 pandemic has forced most researchers to conduct online surveys, and researchers have little control over respondent behaviour. Although the study's inclusion criteria were clearly defined, there was no way to ensure that all the participants met those criteria. There is also the additional limitation inherent with all online surveys – the sample does not represent the general population. Online COVID-19 pandemic research participants tend more often than not to be female and have a higher level of education than the national mean.^40^ On the other hand, the study has a large cohort, and the sample very closely resembled the general population in terms of COVID-19-related variables. Another limitation is that the present study did not assess vaccine opposition in sufficient detail to permit examination of the (high) correlation between mask and vaccine opposition. Our design did not allow us to conclude if mask or vaccine opposition led to higher rates of COVID-19 infection among our respondents; however, it is clear that opposition to masks or vaccines is associated with a low level of adherence to preventive measures in general, which is likely to have increased risks for COVID-19 infection.

### Implications for policy and future research

One of the main conclusions of this study is that very few of our respondents (3.3%) were openly opposed to wearing masks (overt vaccine opposition was also very low, at 3.8%). The severity of the COVID-19 pandemic, however, combined with high political polarisation in most countries, leads mask (and vaccine) opposition to be perceived as a uniform and widespread movement, which may not be the case. The present findings may lead to a greater understanding of the many reasons why some individuals oppose mask-wearing. Such understanding might lead to targeted interventions to decrease opposition to preventive measures. Finally, although the present study focused on opposition to mask-wearing and did not examine vaccination-related factors in detail, the gender difference in terms of opposition to masks versus vaccines (mask opposition was dominated by males, vaccine opposition was not) is important and warrants further research.

It is important to stress that mask opposition is not a sign of mental illness: in hierarchical regression analyses, although claustrophobia symptoms showed predictions that were over and above that of others, variables more closely linked to psychopathology (past psychiatric illness, contact with mental health services) did not show any prediction of mask-related attitude or behaviour. Still, there are several psychological factors that are shared by those who oppose wearing masks. Determining the extent of negative consequences of mask opposition, in terms of COVID-19-related morbidity and mortality, are beyond the scope of this paper. It is, however, worth examining how psychological factors can be modified to increase adherence to measures, since we now know that both vaccines and mask-wearing have been effective in limiting the spread of the pandemic. The present findings indicate that low COVID-19-related anxiety is linked to lower adherence. Further research is needed to determine if this correlation is a result of a psychological factor that we did not measure here. Further, claustrophobia might be responsible for low-level adherence to mask-wearing, although avoidance was more pronounced than low adherence. Future studies should likewise examine the prevalence of needle phobia among those opposed to vaccines. As phobias are relatively easy to treat, mental health professionals may be able to make a significant contribution to the fight against the COVID-19 pandemic.

## Data Availability

The data that support the findings of this study are available upon reasonable request from the corresponding author, C.K., with the permission of Hacettepe University.

## Supplementary material

For supplementary material accompanying this paper visit https://doi.org/10.1192/bjo.2022.603. click here to view supplementary material
