# Supplementary material for: Psychological factors responsible for low adherence to mask-wearing measures during the COVID-19 pandemic
Source: BJPsych Open. 2022 Nov 23;8(6):e203. doi: 10.1192/bjo.2022.603 (PMC9744457; doi:10.1192/bjo.2022.603)
Supplement: Supplementary file 1 [file S2056472422006032sup001.docx]

**Supplement**

**Measures used in the paper “Psychological factors responsible for low adherence to mask-wearing measures during the Covid-19 pandemic”**

| COVID ANXIETY SCALE (CAS) |
| --- |
| To what extent did the following negative consequences/possibilities worry you in the LAST MONTH? |
| Response scale: 0=None, 1=a little, 2=moderately, 3=a lot |
| 1. The possibility of contracting Covid-19 yourself (how worrying has it been in the LAST MONTH)? |
| 2. Possibility of your relatives contracting Covid-19 (how worrying has it been in the LAST MONTH)? |
| 3. The possibility of dying from Covid-19 (how worrying has it been in the LAST MONTH)? |
| 4. The possibility of your loved ones dying from Covid-19 (how worrying has it been in the LAST MONTH)? |
| 5. The possibility of infecting your relatives with Covid-19 (how worrying has it been in the LAST MONTH)? |
| 6. The possibility of losing your job (how worrying has it been in the LAST MONTH)? |
| 7. The possibility of lack of food (how worrying has it been in the LAST MONTH)? |
| 8. The possibility of contracting Covid-19 and not being able to receive treatment (how worrying has it been in the LAST MONTH)? |
| 9. The possibility of another urgent medical need and not being able to get treatment (how worrying has it been in the LAST MONTH)? |

| NEGATIVE ATTITUDES TOWARDS MASK-WEARING (NAM) |
| --- |
| *Below are the reasons some people put forward for not wanting to wear a mask. Did these reasons have an impact on whether you wear a mask (whether you want to or not)? Please respond thinking about LAST MONTH.* |
| Response scale: 2=I agree, that's why I don't want to wear a mask, 1=I agree, but it doesn't affect my wearing a mask, 0=I disagree/doesn't affect my wearing a mask |
| 1.Covid-19 is not as contagious as claimed. |
| 2.Mask makes breathing difficult or reduces oxygen. |
| 3. The mask makes it very hot and makes me sweat. |
| 4.The epidemic is not as dangerous as claimed. |
| 5. The smell of the mask is annoying. |
| 6. Mask gives me a headache. |
| 7. Mask causes itches. |
| 8. It makes me nauseous. |
| 9. The mask causes acnes on my face. |
| 10. Mask breaks makeup. |
| 11. The person wearing the mask touches his face more often, increasing the chance of getting a virus. |
| 12.Mask hurts one's nose and ears. |
| 13. Mask makes people look funny. |
| 14. It is very difficult to wear glasses with a mask; immediately steaming. |
| 15. Masks are not protective. |
| 16. Mask distorts one's appearance. |
| 17. Masks on the market are poor quality. |
| 18. No need to wear a mask outdoors. |
| 19. I can’t afford buying masks. |
| 20. Mask restricts one's freedom. |
| 21. The government should pay for the mask, but it does not |
| 22. It's hard to get used to wearing a mask. |
| 23. Those with a strong constitution do not need to wear a mask. |
| 24. It is difficult to do sports with a mask on. |
| 25. It's hard to focus on a task with a mask on. |
| 26. It's hard to walk with a mask. |
| 27. It’s hard to understand someone talking with a mask on. |
| 28. It's hard to work with a mask on. |
| 29. It's not right to force it. |
| 30. Mask is an excuse to make some people rich. |
| 31. Mask causes rebreathing of polluted air. |
| 32. There are a lot of people who don't wear a mask. |
| 33. Tired of wearing it for a long time. |
| 34. Covid survivors do not need to wear masks. |
| 35. Those who are vaccinated do not need to wear masks. |
| MASK AVOIDANCE SCALE (MAS) |
| *Tick ​​YES if you do it because you don't want to wear a mask, tick NO if it's related to the epidemic.* |
| Response scale: 0=no, 1=yes |
| 1. I have reduced or stopped my out-of-town trips in the LAST MONTH just to avoid wearing a mask. |
| 2. I have reduced or stopped using public transport (minibus, bus) in the LAST MONTH just to avoid wearing a mask. |
| 3. I've been shopping less or quit in the LAST MONTH just to avoid wearing a mask. |
| 4. I've been exercising or walking less, or quit in the LAST MONTH just to avoid wearing a mask. |
| 5. I've been going to work less (or working from home) in the LAST MONTH just to avoid wearing a mask. |
| 6. I have reduced or stopped eating out in the LAST MONTH just to avoid wearing a mask. |

| CLAUSTROPHOBIA SEVERITY SCALE (CSS) |
| --- |
| *Some people feel extreme fear or distress in closed places, in environments where they cannot go out whenever they want, and they avoid such situations. If being in such situations gives you EXTREME fright and distress, or if they are VERY DIFFICULT to enter, tick "DIFFICULT"; otherwise, tick "NOT DIFFICULT". If you think “I have never been in that situation”, imagine “how would I feel if I were in such a situation” and respond accordingly. If your difficulty is only related to the epidemic, tick " NOT DIFFICULT ".* |
| Response scale: 0=not difficult, 1= difficult |
| 1. Get on the elevator. |
| 2.Take the subway. |
| 3.Getting on a crowded bus, minibus. |
| 4. Going to crowded market places, shopping malls. |
| 5. Have an MRI. |
| 6. Pulling the duvet over your face |
| 7. Wearing a turtleneck. |
| 8. Sitting in a windowless room. |
| 9. Sitting in the (hard to escape) bottom seat on the bus or in the movie theatre. |
| 10. Wearing clothes, accessories, masks that cover the face. |
| 11. Locking the door while in the bathroom. |
| 12. Diving underwater while swimming. |
| 13.Traffic jam. |
| 14. Entering a tunnel while traveling. |
| 15. Wearing neck-wrapped necklaces, scarves, etc. |
| 16. Wearing a seat belt in the car. |
| 17. Getting on a plane. |
| 18. Sitting in a locked room. |
| 19. Closed windows. |
| 20. Passing through revolving doors. |
| 21. Being in rooms or hallways with low-ceilings. |
